# A clinical, radiological and isokinetic evaluation in patients with recurrent patellar dislocation undergoing MPFL reconstruction according to Avikainen: a prospective study evaluating early degenerative changes after a minimum 10-year follow-up period

**DOI:** 10.1186/s12891-023-06249-5

**Published:** 2023-02-24

**Authors:** Krzysztof Małecki, Kryspin Niedzielski, Agnieszka Korczyc-Stępnicka, Wojciech Stelmach, Jacek Beczkowski, Jarosław Fabiś, Anna Fabiś-Strobin

**Affiliations:** 1 Clinic of Paediatric Orthopaedics and Traumatology, Polish Mother’s Memorial Hospital Research Institute, Rzgowska 281/289, 93–338 Lodz, Poland; 2 FMC Medical Center, Lodz, Poland; 3 Department of Arthroscopy, Minimally Invasive Surgery and Sports Traumatology, Medical University of Lodz, Lodz, Poland

**Keywords:** Recurrent patellar dislocation, Degenerative changes, Isokinetics

## Abstract

**Background:**

The aim of the study was to conduct a comprehensive functional and radiological follow-up assessment in patients at least 10 years after adductor magnus MPFL reconstruction, and to assess the presence of early degenerative changes.

**Methods:**

The mean age at the time of surgery was 16 years (range: 8 to 18 years, SD 2.5). The follow-up examination was performed at least 10 years following adductor magnus MPFL reconstruction (mean 11 years). Twenty-one patients (26 operated knees) attended the follow-up. The mean age at follow-up was 25.1 years (range 20–29 years).

**Results:**

The significant improvement observed at 3 years, indicated by the Kujala and Lysholm scores, was maintained after 10 years of follow-up (*p* < 0.001). A single recurrence of dislocation was noted in three patients. A significant improvement in radiological parameters was noted. No significant difference in the incidence of chondromalacia, of any degree, was observed compared to controls.

Significantly higher quadriceps peak torque was noted for both angular velocities (60 and 180°/sec) compared to the preoperative readings (*p* < 0.001). Knee flexors were found to be significantly stronger at both 60 and 180°/sec at 10 years follow-up examination (*p* = 0.008 and *p* < 0.001 respectively).

**Conclusion:**

The use of MPFL reconstruction according to Avikainen yields improvements in clinical and radiological results which are maintained throughout the observation period. No significantly greater articular cartilage degeneration was noted in patients after surgical treatment for recurrent patellar dislocation compared to healthy peers.

**Trial registration:**

Registered on Clinical Trails.gov with ID: PMMHRI-BCO.67/2021-A.

## Background

Few studies have examined the long-term results of treating recurrent patellar dislocation in children and adolescents, and none include any long-term assessment of early degenerative changes or the objective performance of extensor apparatus. In addition to patella stability, long-term postoperative degenerative changes may have a significant influence on knee function, patient satisfaction and the final treatment outcome, and the knee flexors performance may display their constitutional weakness in patients with recurrent patellar dislocation, as discussed in the literature [1].

Although a growing range of surgical techniques, are being developed to address such anatomical disorders, the gold standard of treatment remains MPFL reconstruction, supplemented with other techniques depending on anatomical abnormalities [2, 3]. However, a particular problem is posed by patients with open growth plates, who cannot be fully anatomically reconstructed with MPFL. Therefore, in 1993, Avikainen proposed using the native femoral attachment of the adductor magnus muscle for MPFL reconstruction, to avoid damaging the growth plates [4, 5]. This technique has also been reported to be effective in patients with closed growth plates [4, 6].

The aim of the study was to conduct a comprehensive functional and radiological follow-up assessment in patients at least 10 years after adductor magnus MPFL reconstruction, and to assess the presence of early degenerative changes. The study is a continuation of the observation of a cohort of patients established in 2010–2012, operated on by the same technique, by a single operator; the assessment was performed postoperatively according to a uniform protocol.

## Methods

Adductor magnus MPFL reconstruction was performed according to Avikainen in 33 patients (20 girls and 13 boys) (39 knees) between 2010 and 2012. The mean age at the time of surgery was 16 years (range: 8 to 18 years, SD 2.5). The follow-up examination was performed at least 10 years following surgery (mean 11 years). From the observed cohort of 33 patients, 21 patients (26 operated knees) attended the follow-up: *n* = 12 did not attend. The mean age at follow-up was 25.1 years (range 20–29 years). This is the second study based on this cohort: the first was conducted at least 3 years after surgery [6].

The inclusion criteria comprised the following: adductor magus MPFL reconstruction performed in recurrent patellar dislocation (at least two dislocations before surgery), no osteochondral fracture, minimum 10-year follow up, completed research protocol, participation in the previous follow-up study performed 3 years after the operation. The exclusion criteria comprised the following: no consent to participate in the study, age over 18 at the time of surgery, lack of a complete study protocol. Neither trochlear dysplasia nor patella alta influenced the treatment or assessment protocol. All patients underwent MPFL reconstruction according to Avikainen, as described at Malecki et al. [6]. Six patients demonstrated excessive patellar lateralisation (patellar shift), and so patellar tendon transposition was performed according to Roux-Goldthwait. In 16 patients with preoperative patellar tilt, lateral release was applied. Orthosis immobilisation was used for 6 weeks. For the first 2 weeks, the orthosis was completely locked in a 10-degree flexion; for the next 2 weeks, a 0–30-degree range of motion was permitted in the orthosis, increasing to 0–60 degrees after another 2 weeks. Full loading of the operated limb was allowed 4 weeks after the procedure. Outpatient rehabilitation was carried out for 6 months post-op. All participants were available for comprehensive evaluation after 3 years post op, as published previously [6]. The loss of 12 patients between follow-ups (i.e. three- and 10-year follow-ups) was not dependent on the researchers. All authors participated in patient examination and data collection. The clinical result was assessed using the Lysholm Knee Scale and the Kujala Anterior Knee Pain Scale [7, 8]. In addition, apprehension test was performed and joint laxity were assessed according to the Beighton scale [9]. At the 10-year follow-up MRI of the knee was performed.

**Fig. 1:**
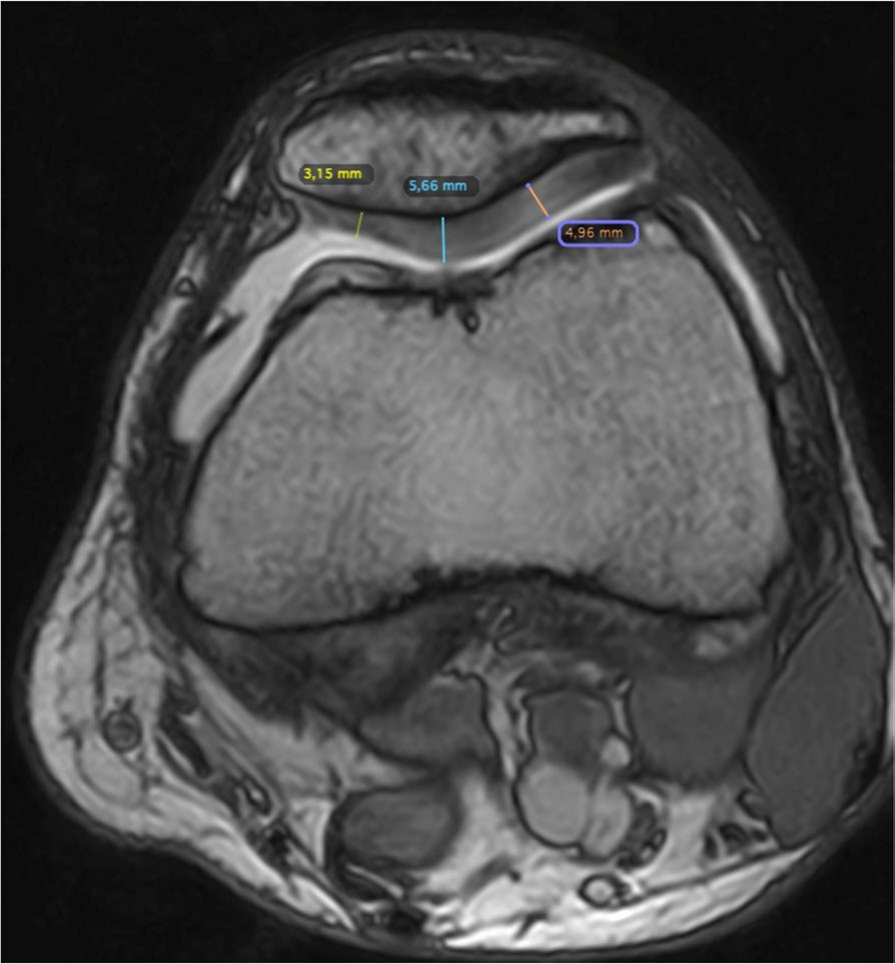
MRI cross-section with reference points for the patellar cartilage measurement

**Fig. 2:**
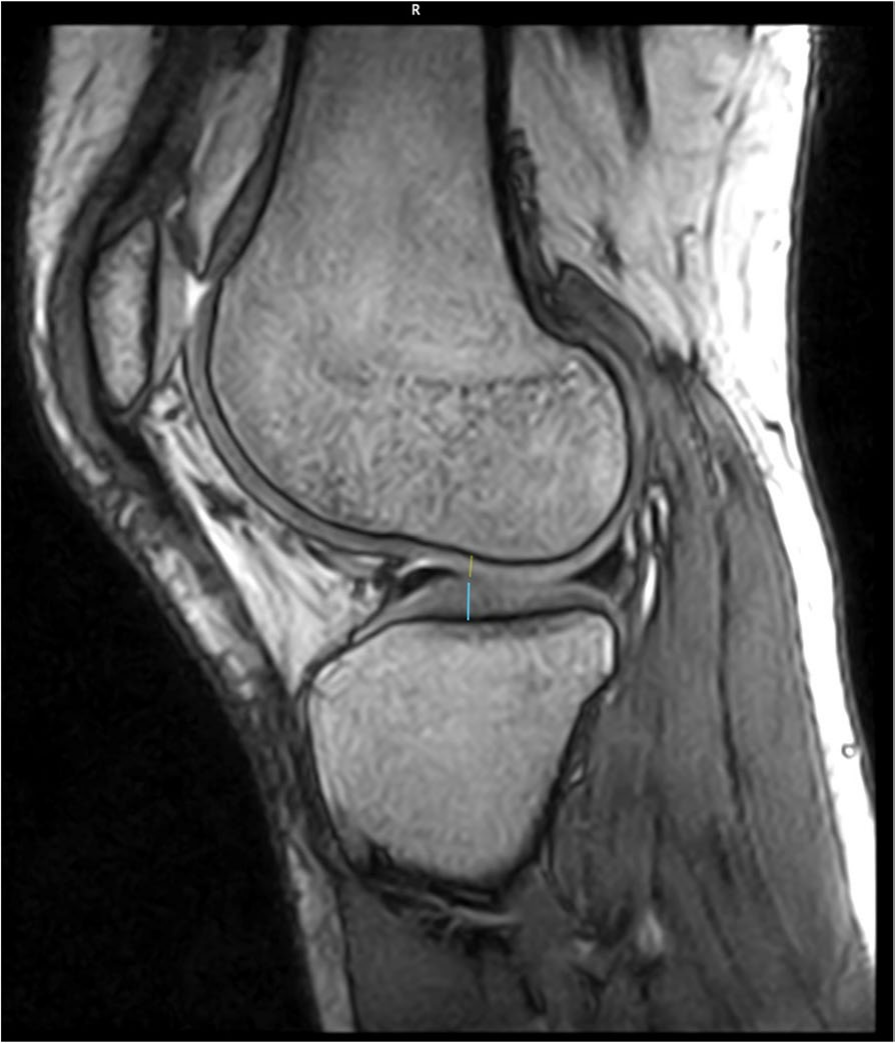
MRI cross-section with reference point for the cartilage measurement of the medial and lateral femoral and tibial condyles (saggital plane)

**Fig. 3:**
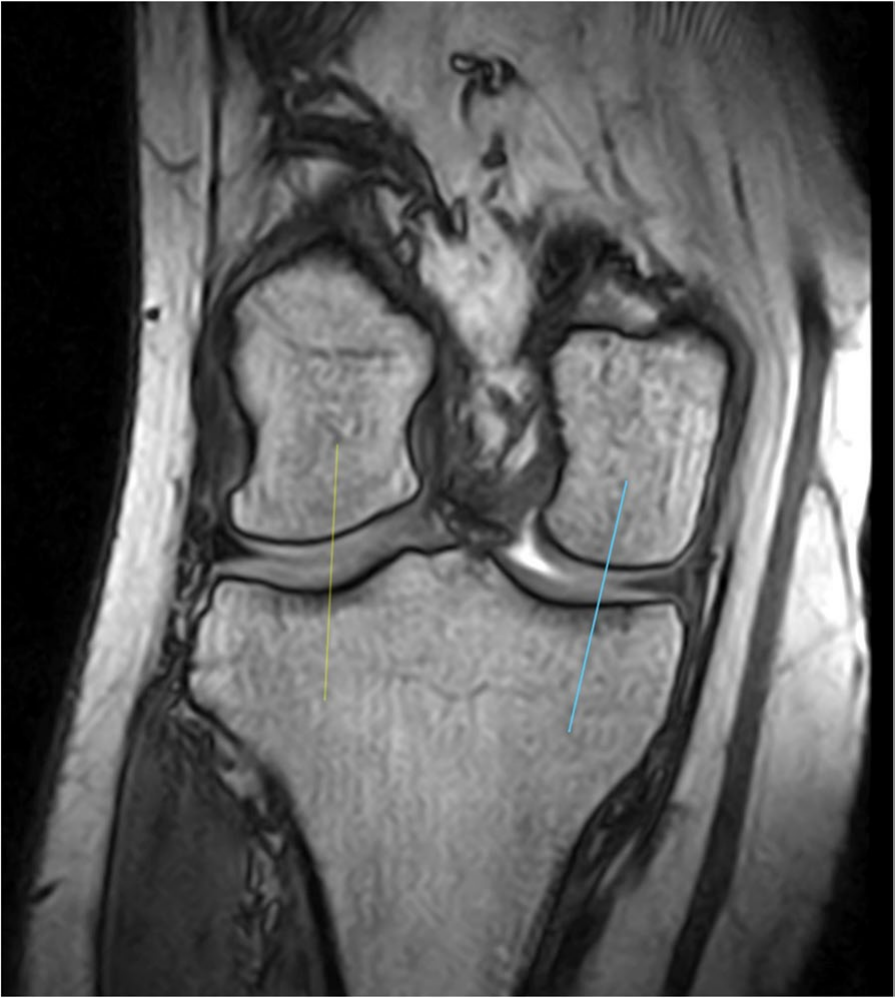
MRI cross-section with reference point for the measurement plane for the loading area of the cartilages of the medial and lateral femoral and tibial condyles (frontal plane)

The radiographic examination revealed the height of the patella according to Caton-Deschamps, patellofemoral angle and congruence angle as described by Malecki et al. [6]. MRI scans were also performed of the knee joints to assess early degenerative changes; the procedure was performed with a field strength of 0.32 T of the O-Scan model (Esaote). The thickness of the articular cartilage was determined based on 1.9 mm-deep images, obtained by the 3D-Sharc sequence, while that of the patellar cartilage was measured by cross-sections at three reference points: Point 1 – the cartilage of the medial surface, Point 2 – the cartilage at the ridge of the articular surface of the patella and Point 3 – the cartilage of the lateral articular surface (Fig. 1). The reference point for the measurement plane for the loading area of the cartilages of the medial and lateral femoral and tibial condyles was located on the bone, at the height of the tendon notch of the popliteus muscle at the lateral femoral condyle; this marked the centre of the condyles in the frontal projection at this level (Figs. 2 and 3) [10]. The degree of chondromalacia was assessed according to the Outerbridge scale [11]. The cartilage thickness values were compared with those from a control group comprising 20 patients aged 20–30 years; this group was created for the purpose of this analysis. Their MRIs revealed no evidence of any abnormalities. The thickness of the patellar articular cartilage was compared between groups at the three separate points and as an overall measurement.

To strengthen the quality of inference, this comparison was based on the ratio of the thickness of the patellar cartilage to its thickness at the reference points of the tibial and femoral condyles. It was calculated as follows:

$$\textrm{Total}\ \textrm{measurements}\ \textrm{from}\ \textrm{the}\ \textrm{patella}/\textrm{Total}\ \textrm{measurements}\ \textrm{from}\ \textrm{the}\ \textrm{femorotibial}\ \textrm{joint}.$$

This calculation was performed for each case from the cohort and control groups to allow a global assessment of articular cartilage in patients with recurrent patellar dislocation, labelled the *cartilage disparity index* for the purposes of this study. A higher index value indicates a smaller disproportion between the thickness of the patellar cartilage and the femorotibial joint.

Quadriceps and hamstring peak torque at 60 and 180°/sec were measured 1 month before and at least 10 years after surgery (Biodex Multi-Joint System-Pro, Biodex Medical Systems, Inc., New York, USA). The results were also compared to those obtained during the previous follow-up, i.e. 3 years after surgery.

The frequencies of the significant differences were compared using the non-parametric Wilcoxon’s test for non-normally distributed data, the t-test for normally-distributed data, and the Mann-Whitney U-test for independent data. The independent chi-square test was also used for a four-field array for categorical data. Statistical significance was assumed for *p* < 0.05. The analysis was performed using the STATISTICA software package, ver. 10 (Statsoft, Inc. 2011, DASS).

The study was approved by the institutional review board of the Polish Mother’s Memorial Hospital (registration No 67/2021) and registered on Clinical Trails.gov with ID: PMMHRI-BCO.67/2021-A. The study was carried out in accordance with World Medical Association Declaration of Helsinki. Informed consent was obtained from all participants.

## Results

### Clinical examination

The significant improvement observed at 3 years, indicated by the Kujala and Lysholm scores, was maintained after 10 years (*p* < 0.001): the results had not deteriorated significantly between follow-ups (Kujala *p* = 0.968 and Lysholm *p* = 0.915). At the current (10-year) follow-up, a single recurrence of dislocation was noted in three patients. Six patients were found to have a positive apprehension test at 10 years, compared to three patients after the three-year follow-up (*p* = 0.538). The mean Beighton score had decreased significantly over the 10 years since the first study (*p* < 0.001) (Table 1).

**Table 1:** Comparison of the Lysholm and Kujala and Beighton scale results

Tested value (points)	pre op (0 y)	3 y post surgery	10 y post surgery
**Lysholm Scale**			
**Mean**	65.9	90.9	90.7
**Range**	51–95	71–100	74–100
**SD**	11.4	10.0	7.3
***p *value (3–10 y)**	–	***p*** **= 0.915**	
***p *value (0–10 y)**	***p*** **< 0.001**		
**Kujala Scale**			
**Mean**	67.0	91.8	91.8
**Range**	48–84	70–100	9.9
**SD**	91.8	72–100	8.9
***p *value (3–10 y)**	**–**	***p*** **= 0.968**	
***p *value (0–10 y)**	***p*** **< 0.001**		
**Beighton Scale**			
**Mean**	4.9	–	3.6
**Range**	0–9		0–9
**SD**	2.4		1.8
***p *value (0–10 y)**	***p*** **< 0.001**		

### Radiological assessment

A significant improvement in radiological parameters was noted, with no deterioration since the three-year follow up (Table 2). No degenerative changes were visible on the radiographs in any of the patients.

**Table 2:** Statistical analysis of radiological assessment

Tested value	Pre op. (0 y)	3 y post-op.	10 y post-op.
**Caton-Deschumps index**			
**Mean**	1.30	1.25	1.18
**Range**	0.8–1.57	0.9–1.90	1.54–0.9
**SD**	0.23	0.22	0.17
***p *value (3–10 y)**	**–**	***p*** **= 0.912**	
***p *value (0–10 y)**	***p*** **= 0.519**		
**Patellofemoral angle (deg)**			
**Mean**	3.46	−5.54	−6.6
**Range**	−8 - 34	−15 - 4	−17 – 3
**SD**	10.4	4.9	5.1
***p *value (3–10 y)**	–	***p*** **= 0.352**	
***p *value (0–10 y)**	***p*** **< 0.001**		
**Congruence angle (deg.)**			
**Mean**	19.3	−4.7	−6.2
**Range**	−15 - 70	−30 - 16	−30 - 14
**SD**	20.5	10.7	12.5
***p *value (3–10 y)**	–	***p*** **= 0.670**	
***p *value (0–10 y)**	***p*** **< 0.001**		
**Trochlear groove angle (deg.)**			
**Mean**	148	147	147
**Range**	140–164	135–160	139–153
**SD**	6.6	6.6	6.2
***p *value (3–10 y)**	–	***p*** **= 0.270**	
***p *value (0–10 y)**	***p*** **= 0.052**		

MRI scans of the cartilage of the patellar joint revealed I degree chonodromalacia (Outerbridge) in six patients, II and III degree in one patient each, and IV degree in two patients. No significant difference in the incidence of chondromalacia, of any degree, was observed compared to controls (Table 3). Lateral compression of the patella was only noted in two cases. No significant thinning was found compared to the control group at any of the reference points for the patella (Table 4). The cartilage disparity index was not significantly lower compared to the control group, indicating no difference between the thickness of the patellar cartilage compared to the femorotibial joint (Table 5).

**Table 3:** Outerbridge MRI classification assessment in both test and control group

	Chondromalacia patellae				
	I∘	II∘	III∘	IV∘	in total
**Test group *n*** **= 26**	23.1% (6/26)	3.8% (1/26)	3.8% (1/26)	7,7% (2/26)	38,4% (10/26)
**Control group *n*** **= 20**	10.0% (2/20)	5.0% (1/20)	5.0% (1/20)	10.0% (2/20)	30.0% (6/20)
***p*-value**	*p* = 0.246	*p* = 0.849	*p* = 0.849	*p* = 0.783	*p* = 0.550

**Table 4:** Comparison of the patellar cartilage thickness in both research and control group

	Average patellar cartilage thickness [mm]							
	Medial		Center		Lateral		Total (average sum)	
	mean	SD	mean	SD	mean	SD	mean	SD
**Test group *n*** **= 26**	3.58	0.95	4.37	1.00	4.02	0.97	11.98	2.48
**Control group *n*** **= 20**	4.37	0.98	4.21	0.87	4.62	0.81	13.05	2.10
***p*-value**	*p* = 0.219		*p* = 0.928		*p* = 0.110		*p* = 0.201

**Table 5:** Results of the cartilage disparity index analysis

	Average of the sum of the measurements of the patella cartilage thickness (3 points) [mm]	Average of the sum of the measurements of the LFC MFC LTC MTC cartilage thickness [mm]	Average of sum quotients (cartilage disparity index)	***p***-value
**Test group**	11.98	10.32	1.16	*p* = 0.704
**Control group**	13.05	11.99	1.09	

### Isokinetic assessment

**Fig. 4:**
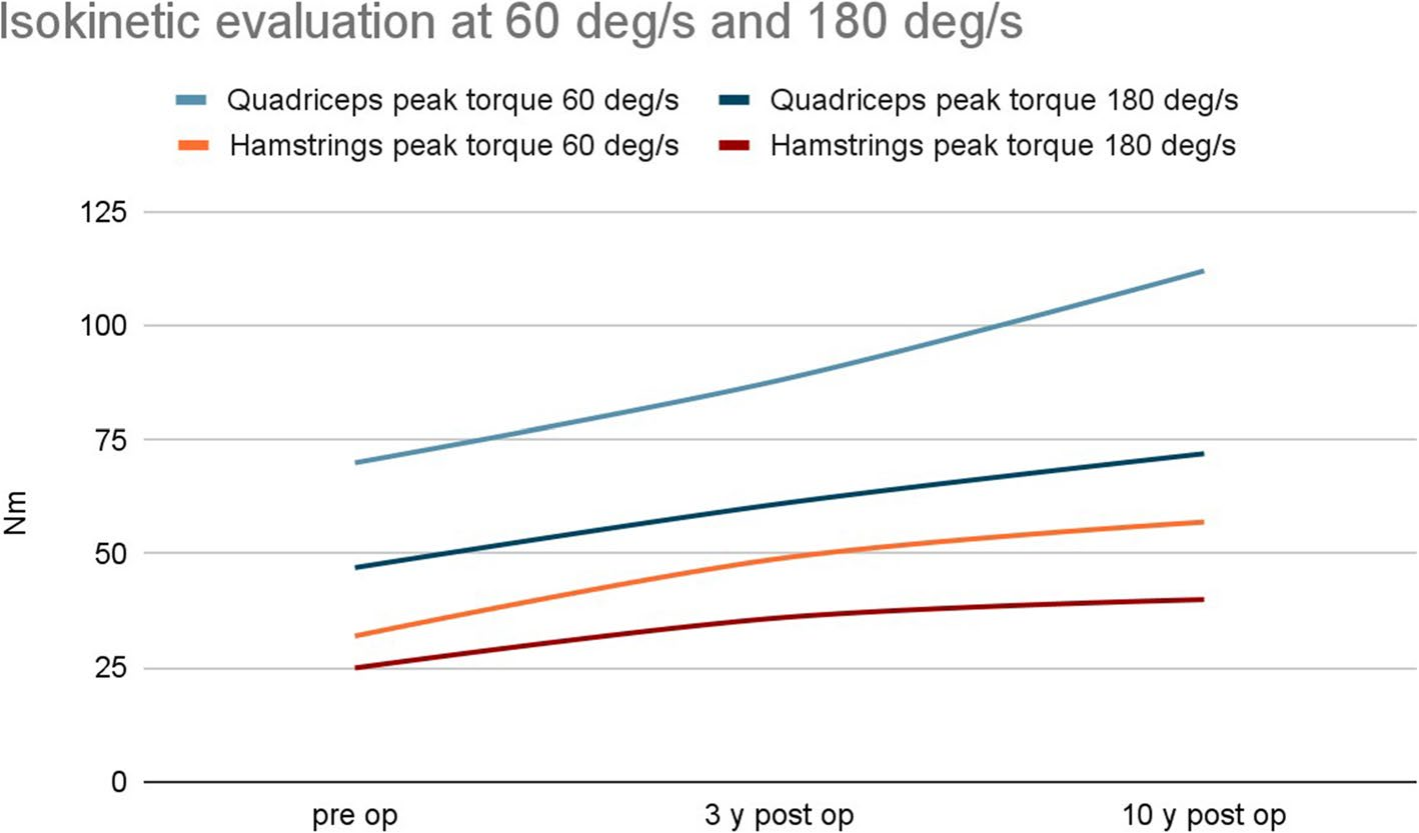
Analysis of the results of isokinetic evaluation at 60 i 180 deg/s – diagram

At the post- 10-year follow-up, significantly higher quadriceps peak torque was noted for both angular velocities (60 and 180°/sec) compared to the preoperative readings and those taken 3 years after the operation (both *p* < 0.001). Knee flexors were found to be significantly stronger at both 60 and 180°/sec at the 10-year follow-up examination (*p* = 0.008 and *p* < 0.001 respectively). However, no further increase in flexor peak torque was noticed between the three-year and 10-year follow-ups for either angular velocity (60 °/sec, *p* = 0.063; 180°/sec, *P* = 0.660) (Table 6, Fig. 4).

**Table 6:** Analysis of the results of isokinetic evaluation at 60 i 180 deg/s

Speed	Pre op (average)	3 y post op	10 y post
**Quadriceps peak torque [Nm]**			
60 deg/s	70	88	112
*p*-value		*p* = 0.004	
	***p*** **< 0.001**		
180 deg/s	47	61	72
*p*-value		*p* = 0.002	
	***p*** **< 0.001**		
**Hamstrings peak torque [Nm]**			
60 deg/s	32	49	57
*p*-value		*p* = 0.063	
	***p*** **= 0.008**		
180 deg/s	25	36	40
		*p* = 0.660	
	***p*** **< 0.001**

## Discussion

The improvement in clinical and radiological parameters observed at the three-year follow-up was found to be maintained throughout the observation period. At the 10-year follow-up, single recurrences of dislocation were noted in three patients, which occurred during sport activities. A rehabilitation program was implemented to prevent further dislocations. In one patient, MPFL reconstruction from the gracilis tendon was performed due to persistent instability. Those receiving conservative treatment for recurrence demonstrated a positive apprehension test; however, in the remaining four patients with a positive apprehension test, no recurrence was noted throughout the follow-up period. The mean Beighton scale score decreased significantly over time, indicating that joint hypermobility decreases with age.

More positively, the radiological examination revealed a significant improvement in the congruence angle and patellofemoral angle, and no significant deterioration was observed since the first follow-up examination, i.e. 3 years after surgery. No degenerative changes were visible on the radiographs in any of the patients. The best tool for such assessment is MRI, as it allows an accurate assessment of the thickness of the cartilage in the entire range of the joint. As we know, the goal of treatment with patellar instability is not only to stabilize the patella, but also to prevent degenerative changes occurring in the patellofemoral joint. Our comparison of the MRI images from the test group and the healthy controls yielded important data which allowed us to accurately assess the severity of degenerative changes 10 years after surgery. The aim was not only to determine the condition of the cartilage in the patellar joint, but to make a more global assessment of the entire knee joint to confirm or negate isolated degenerative changes in the anterior knee compartment. However, it is important to note that any instability of the patella, the associated surgical treatment and the potential rotational instability in the femorotibial joint may accelerate the overall degradation of the articular cartilage, and not only for the patellofemoral joint.

Our analysis did not reveal any statistically significant differences in articular cartilage composition compared to the control group. This result is at least satisfactory. In addition, no significant differences in incidence based on the Outerbridge scale were found between groups, which confirms our quantitative analysis of the articular cartilage. However, due to a lack of similar research in this area, it is not possible to compare these findings with previous studies.

Interestingly, the isokinetic analysis indicated a gradual increase in the strength of the quadriceps over the 10-year observation period. This is probably related to natural growth and weight gain among the patients, but it may also indicate that the patella remained stable, thus allowing the proper functioning of the quadriceps muscle. Interestingly, no further increase in flexor strength was observed after the three-year follow-up examination. It is difficult to provide a clear reason for this, but the data could confirm that patients with patellar instability demonstrate constitutional weakening of the knee flexors: we would expect flexor strength to gradually increase with quadriceps strength [1, 12].

The present study is the first to make a broad assessment of treatment results based on isokinetic assessment of muscle performance and MRI assessment of degenerative changes over such a long period. Although some studies have used long-term follow-ups of patients operated on for recurrent patellar dislocation in adolescence, these used highly-heterogeneous groups with regard to inter alia age; in addition, none examined muscle performance at a follow-up period of at least 10 years. Furthermore, while some studies have employed longer follow-ups, these demonstrate considerable heterogeneity with regard to inter alia classification of the treatment result, operating method and age group.

An X-ray study by Maenpaa et al. describes the occurrence of degenerative changes in patients with patellar instability after a mean follow-up period of 13 years; osteoarthritis was noted in 17% of operated cases of recurrent dislocation and in 12% of unoperated cases. This may indicate that the operation itself does not delay the onset of degenerative changes [13].

In Nomura et al. MPFL reconstruction surgery was found to yield 88% good results with two recurrences of dislocation in 24 adult knees at a 12-year follow-up. In addition, three knees were found to demonstrate moderate osteoarthritis, including two with clear progression of osteoarthritic changes [14].

A X-ray study of a cohort of skeletally-immature patients with first-time dislocation of the patella (*n* = 232) by Sanders et al. found 20% to display symptomatic osteoarthritis. Of these, 104 experienced recurrence within a mean follow-up period of 12 years [15].

A significant study in the treatment of recurrent patellar dislocation is that of Arnbjornsson et al., who evaluated 29 adult patients with bilateral recurrent patellar dislocation, of which one knee received surgical treatment. Over a 14-year follow-up period, the unoperated knees demonstrated better results according to Lysholm, while the operated group revealed a higher percentage of degenerative changes. However, none of the patients underwent MPFL reconstruction: all received soft tissue proximal realignment or distal realignment tibial tuberosity medialisation, or both [16].

Bengtsson et al. assessed changes in articular cartilage of 16 young adults over a mean follow-up period of 8.5 years after first dislocation. Post-contrast evaluation revealed qualitative differences between the superficial part of the patellar articular cartilage and the healthy side; however, no correlation was observed between the changes and clinical status. The authors also noted a slight weakening of the quadriceps, which also did not correlate with the cartilage assessment on MRI [17].

A study of 129 patients by Vollenberg et al. found chondral lesions to be present in 71% cases of acute dislocation, 82% of recurrent dislocation and 97% of chronic dislocation. It was proposed that the frequency of dislocations and the degree of instability influence the prevalence of degenerative changes, and this should be considered in surgical decisions [18].

Many studies describe the treatment of recurrent patellar dislocation with MPFL reconstruction, which is regarded as the method of choice. A systematic review by D’Ambrosi et al. examined the recurrence of dislocation and its complications in patients under 20 years of age treated with MPFL reconstruction. With a median follow-up of 30 months, and a median age at surgery of 14 years, the overall performance was good, with only 7% demonstrating instability after surgery. Unfortunately, few relevant works are available for comparison, including systemic reviews, and many use a short follow-up with no functional assessment or MRI [19].

One potentially valuable alternative to MPFL reconstruction involves the use of trochleoplasty, but this method is reserved for skeletally-mature patients with trochlear dysplasia. The procedure was subjected to a 12-year observational study by Rouanet et al. in a heterogeneous group of knees with regard to age and morphology; of the initial group, 20% demonstrated failures requiring reoperation. In addition, six knees underwent arthroplasty due to pain and stage IV osteoarthritis [20]. Von Knoch et al. report degenerative changes to be present in 30% of 45 knees over a mean eight-year follow-up period following trochleoplasty, with no recurrence of dislocation. The patients were operated at age 15–31 years [21].

A study by Puijk et al. including the four-in-one soft tissue procedure in 34 operated knees from skeletally-mature and -immature patients found 23.5% to demonstrate recurrent dislocation over a mean follow-up period of 10 years. In addition, the functional outcomes were rather unsatisfactory, i.e. 81 points according to Kujala and 76 points according to Lysholm [22].

Our present findings, and those of the studies cited above, confirm the value of MPFL reconstruction as the method of choice in recurrent patellar dislocation, resulting in a low percentage of cartilage degenerative changes over a long follow-up period while maintaining good clinical results. Distal realignment techniques, such as Roux-Goldthwait, Grammont and Fulkerson, should be used as supplementary treatments in cases where it is necessary to change the patellar tracking. Trochleoplasty should be limited to carefully-selected cases of trochlear dysplasia in skeletally-mature patients.

A key limitation of our study is that it is based on a relatively small group of patients, and so care should be taken when interpreting our data. However, as our findings are based on a 10-year follow-up period, and include isokinetic and MRI analyses, they nevertheless represent a significant contribution to this field.

## Conclusion

The use of MPFL reconstruction according to Avikainen yields improvements in clinical and radiological results which are maintained throughout the observation period. Quadriceps strength was found to increase throughout the 10-year observation period, but no such growth was observed in the case of flexors. This confirms previous conclusions indicating a significant weakening of the knee flexors in patients with patellar instability. No significantly greater articular cartilage degeneration was noted in patients after surgical treatment for recurrent patellar dislocation compared to healthy peers.

## Data Availability

Data supporting the findings of this study are available from the corresponding author [KM] upon reasonable request.
